# Butterflies of the high-altitude Atacama Desert: habitat use and conservation

**DOI:** 10.3389/fgene.2014.00334

**Published:** 2014-09-24

**Authors:** Emma Despland

**Affiliations:** Department of Biology, Concordia UniversityMontréal, QC Canada

**Keywords:** Lepidoptera, conservation, Chile, Andes, species abundance, distribution

## Abstract

The butterfly fauna of the high-altitude desert of Northern Chile, though depauperate, shows high endemism, is poorly known and is of considerable conservation concern. This study surveys butterflies along the Andean slope between 2400 and 5000 m asl (prepuna, puna and Andean steppe habitats) as well as in high and low-altitude wetlands and in the neoriparian vegetation of agricultural sites. We also include historical sightings from museum records. We compare abundances between altitudes, between natural and impacted sites, as well as between two sampling years with different precipitation regimes. The results confirm high altitudinal turnover and show greatest similarity between wetland and slope faunas at similar altitudes. Results also underscore vulnerability to weather fluctuations, particularly in the more arid low-altitude sites, where abundances were much lower in the low precipitation sampling season and several species were not observed at all. Finally, we show that some species have shifted to the neoriparian vegetation of the agricultural landscape, whereas others were only observed in less impacted habitats dominated by native plants. These results suggest that acclimation to novel habitats depends on larval host plant use. The traditional agricultural environment can provide habitat for many, but not all, native butterfly species, but an estimation of the value of these habitats requires better understanding of butterfly life history strategies and relationships with host plants.

## INTRODUCTION

The butterfly fauna of the high-altitude Atacama desert is depauperate in terms of species diversity and abundance, but nonetheless of conservation interest due to high endemism, high species turnover along both latitudinal and elevational gradients (Samaniego and Marquet, 2009), as well as narrow phenological windows of activity spread across the austral spring and summer (Despland et al., 2012).

Although butterfly species in this region are well described (Peña and Ugarte, 1996), in most cases, little or nothing is known of their life-cycle, phenology or habitat use. This ignorance is exacerbated by unsystematic sampling, in variable and often inappropriate seasons (Shapiro, 1992), combined with often narrow phenological windows of activity: systematic biweekly sampling showed some species were only active during a few weeks, with these windows spread between October and May (Despland et al., 2012). Much therefore remains to be explained about the distributions and host plant associations of Andean butterflies (Shapiro, 1992), at a time when these species are under increasing threat from climate change and habitat loss (RIDES, 2003).

Documenting, describing and understanding this diversity remains an important challenge in biogeography and conservation biology (PDMAT, 2008). The present study examines patterns of habitat use by high-altitude desert butterflies and relates them to conservation issues through a comparison between native and agricultural habitats and between wet and dry years.

**FIGURE 1:**
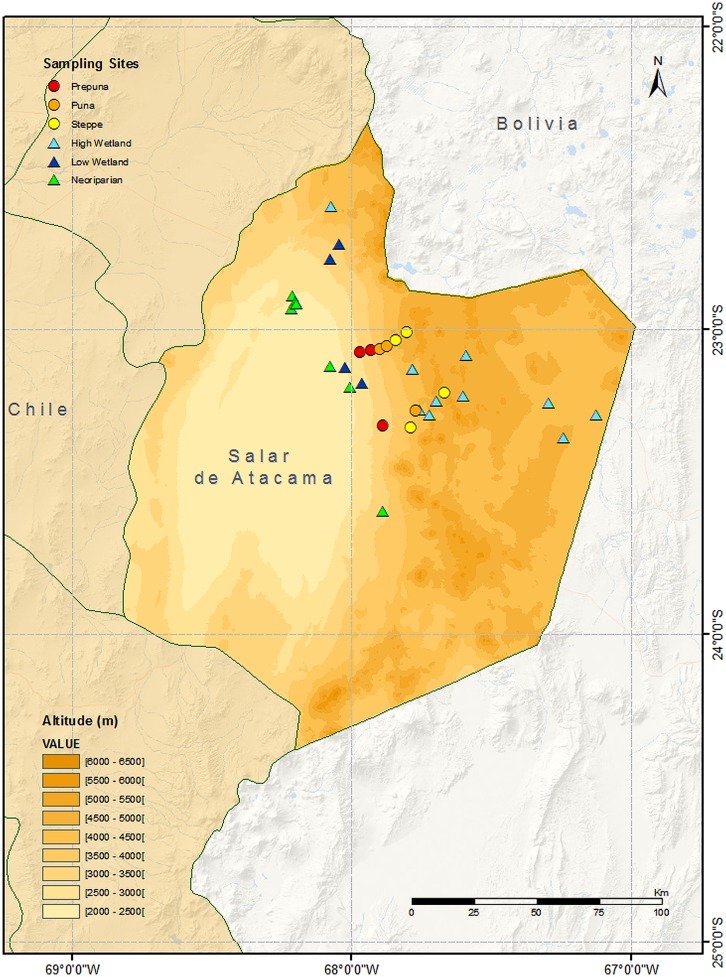
**Topographical map showing sample locations (including modern and museum specimens) by habitat type**.

In the desert Andes of Northern Chile (see **Figure 1**), the region between the salar de Atacama and the altiplano is characterized by slope vegetation divided in clear altitudinal belts (prepuna, puna and Andean steppe) up to the Andean summits and by islands of wetland habitat in areas with surface or near-surface waters (Marquet et al., 1998; Jaksic et al., 1999). This region is considered a zone of conservation priority (Cavieres et al., 2002), due to the high level of endemism of its flora [34–56% (Squeo et al., 1998)]. Studies also suggest high local levels of endemism among beetles (Jerez, 2000) and insects generally (Marquet et al., 1998).

This zone is also under pressure from increasing human use, and desiccation is increasingly severe on an already arid landscape (RIDES, 2003). Butterflies are under increasing anthropic threat, primarily through habitat loss (PDMAT, 2008). In the Chilean Andes generally, several butterfly species are recorded as locally extirpated or generally threatened due to unregulated livestock grazing and concomitant habitat loss (Benyamini, 1995; Johnson, 1999, 2000). In the Atacama Desert, grazing pressure on Andean slopes and high-altitude wetlands destroys host plants (Benyamini, 1995), whereas most low-altitude wetlands are heavily impacted by human settlement and increasingly dominated by agricultural and exotic plants (Gutierrez et al., 1998). Several species, recorded from the area in the past, have not been seen in recent years, raising concerns about their conservation status (Johnson, 1999).

The purpose of this study is to characterize the butterfly fauna of the Andean Pacific slope in the Atacama Desert of Northern Chile and to study patterns of distribution of butterflies across habitats, including Andean slope habitats along an altitudinal gradient, high and low-altitude wetlands and the neoriparian vegetation in inhabited oases. An analysis of butterfly species richness along a slope altitudinal gradient in this region shows a mid-altitude peak in diversity in the puna ecozone where plant productivity is highest (Despland et al., 2012). The present study study compiles data from several recent surveys and from historical museum specimens, to compare these Andean slope faunas with those of wetlands, to compare faunas between wet and dry years and to examine which species persist in the neoriparian plant communities of heavily impacted oases.

## MATERIALS AND METHODS

### DATA SOURCES

We gathered data from a range of different sources, including recent surveys and historical museum collections, from the zone around the salar de Atacama (2400 m asl) up the Pacific slope of the Andes to the altiplano (ca. 5000 m asl) between 22^o^50′–23^o^50′ S and 67^o^45′–68^o^15′ W (see **Figure 1**).

Systematic sampling was conducted over the austral spring to autumn (October to June) in the years 2008–2009 and 2009–2010. Observations were conducted between 1100 and 1500 h for a period of 15 min at each site. Biweekly sampling was conducted along an altitudinal transect across the prepuna (two sites), puna (two sites) and Andean steppe (two sites) slope habitats (Despland et al., 2012), and at two sites within San Pedro de Atacama (neoriparian vegetation). During the austral summer (January to March) biweekly sampling occurred in low level wetlands (two sites) and neoriparian zones (three sites) as part of an outreach project with local indigenous schoolchildren [Descubriendo Nuestra Biodiversidad project (Barros et al., 2009)]. Several expeditions were conducted to four high-altitude wetlands during the austral summer (**Figure 1**).

Historical specimens were also consulted in the collections of the Museo Nacional de Historia Natural, the Universidad Metropolitana de Ciencias de la Educación, and the private collection of Dr. Pedro Vidal. Additional records were also obtained from the Darwin Institute Mariposas Andinas project and from Dr. Gerardo Lamas (Museo de Historia Natural, Universidad Mayor de San Marcos, Lima). Most records date from the 1920–1970s.

### HABITATS

The opposing temperature and humidity gradients along the Andean slope lead to the formation of well-defined altitudinal vegetation belts (Villagran et al., 1981), with clearly defined prepuna, puna and Andean steppe communities (Despland et al., 2012). Wetlands occur locally where surface waters are present and constitute habitat islands with a distinct and more diverse flora – we distinguish between high- and low-altitude wetlands based on differences in plant communities: dominated by grasses and cushion plants above 3500 m asl, and by trees, shrubs, grasses, and annuals below (Squeo et al., 2006b).

Human settlements around the salar de Atacama are concentrated around lower altitude oases and riparian areas, and as a result these are often dominated by introduced plants, including agricultural crops, ornamentals and ruderal species (Gutierrez et al., 1998). Similarly, in arid zones of Argentina, steppe communities are constituted of native austral species, but river valleys are dominated by exotics, mostly of European origin: this neoriparian vegetation can be considered a distinctive plant community, which has been colonized by a subset of the regional butterfly fauna (Shapiro, 2002). In our study, we therefore distinguish between low-level wetlands dominated by native vegetation and neoriparian sites dominated by human activity and exotic vegetation. Slope communities and high-altitude wetlands are constituted almost exclusively of native species.

Sampling sites were categorized by habitat type, according to the vegetation, as follows:

1 Prepuna (2500–3500 m asl): slope habitats at the base of the mountains, in the hottest and driest zone with precipitation averaging <50 mm per year (Schmidt, 1999). Vegetation is concentrated in dry riverbeds, dominated by xerophyllic shrubs and cactus (two sites sampled in 2008–2010 and one recorded from museum specimens).
2 Puna (3500–4200 m asl): mid-altitude slope habitat of highest plant cover and diversity. The vegetation is dominated by shrubs, with tussock grasses and cacti (two contemporary and two historical sites).
3 Andean Steppe (4200–500 m asl): where the Andean slope reaches the altiplano, the vegetation is dominated by tussock grasses and cushion plants with annuals appearing in the austral spring. At this altitude, summer monsoon precipitation is more abundant (around 250 mm per year), mostly falling as snow, which sublimates under high daytime temperatures (two contemporary and one historical site).
4 High-Altitude Wetlands (above 3500 m asl): vegetation is dominated by cushion plants and grasses. These habitat islands have higher plant productivity and diversity than surrounding steppe habitat, and contain many endemic species. They constitute staging areas for migratory birds and breeding habitat for birds and mammals, and have been traditionally used as pasture for camelids (Squeo et al., 2006b). There is concern that these could be diminishing due to water extraction associated with mining (RAMSAR, 1981; RIDES, 2003; four contemporary and nine historical sites).
5 Low-Altitude Wetlands: This category includes only those surface water sites with minimal human intervention that are still dominated by native vegetation, including trees, shrubs, herbs, and grasses (three contemporary and two historical sites).
6 Neoriparian Vegetation: Agricultural areas, dominated by corn, alfafa and fruit trees grown under irrigation, as well as invasive plants, some native ruderal herbs and grasses and native trees and shrubs (three contemporary and four historical sites). This neoriparian community is typical of traditional indigenous agriculture (Marquet et al., 1998) and is widely distributed in oases and riparian habitats of the Atacama Desert (Gutierrez et al., 1998).

A map was constructed showing modern sampling sites and locations for museum specimens, classified by habitat type (see **Figure 1**).

### ANALYSIS

Observations from 2008 to 2010 were standardized to an abundance index representing the average number of individuals observed in a 15 min interval for use in the cluster analysis and *t*-tests. For inclusion of museum specimens (for which information on sampling time is absent) in the cluster analysis, each specimen was considered to represent a 15 min period.

We compared community composition between habitats using the Morisita-Horn abundance-based similarity estimator, then grouped habitats by community similarity using single-linkage cluster analysis and displayed the result as a dendrogram (Sanders et al., 2003).

Communities were also compared between the two sampling years, to investigate weather effects on butterfly distribution and abundance. Precipitation, and hence water availability, differed considerably between the 2 years of study: precipitation records from the nearest weather station (Calama El Loa, 22^o^29′S; 68^o^54′ W) indicate 5.1 mm precipitation in January–March 2009 but only 0.9 mm in January–March 2010. No precipitation at all was recorded in the other months of the year. Numbers of butterflies for each species per month were compared between years using paired *t*-tests for each of the habitat types. Statistical analyses were conducted using the R software environment.

## RESULTS

### BUTTERFLY COMMUNITIES

A total of 17 species from the families Hesperiidae, Pieridae, Lycaenidae and Nymphalidae were observed, from live observations of 608 individuals and 145 museum specimens. Specimens of all species collected have been placed in the Museo Nacional de Historia Natural in Santiago (Chile). The list of species, with their abundance in different habitats is shown in **Table 1**. In general, historical records showed similar faunas in similar habitats as modern observations. One notable exception is the white *Infraphulia illimani* for which one record exists from a high-altitude wetland in 1967, but which was not observed in modern expeditions.

**Table 1:** Species list and abundance of butterflies observed in the different habitats.

**Species**	**High wetlands**	**Steppe**	**Puna**	**Prepuna**	**Low wetlands**	**Neoriparian**
*Pyrgus barrosi* (Ureta, 1956)	(1)	**0.10**				
*Pyrgus fides* (Hayward, 1940)					(1) **0.01**	(1) **0.09**
*Hylephila isonira isonira* (Dyar, 1913)					(17) **2.42**	(20) **4.84**
*Hylephila boulleti* (Mabille, 1906)	(23) **1.20**					
*Colias flaveola blameyi* (Jörgensen, 1916)	**0.20**					
*Hypsochila wagenknechti sulfurodice* (Ureta, 1955)			**1.20**	**0.18**		
*Infraphulia illimani* (Weymer, 1890)	(1)					
*Phulia nymphula nymphula* (Blanchard, 1852)	(21) **0.80**		(1) **0.10**			
*Pierphulia rosea rosea* (Ureta, 1956)	**0.09**	(10) **1.10**				
*Tatochila mercedis macrodice* (Staudinger, 1899)			(5) **0.10**		(1) **0.03**	
*Madeleinea pelorias* (Weymer, 1890)	(1)		(6) **0.70**			
*Nabokovia faga* (Dognin, 1895)						**0.02**
*Strymon flavaria* (Ureta, 1956)				(1) **0.50**		
*Leptotes trigemmatus* (Butler, 1881)				**0.18**	(1) **1.42**	(12) **5.31**
*Faunula leucoglene leucoglene* (Felder and Felder, 1867)	(14) **2.80**	**1.90**	**0.80**			
*Palmaris penai* (Hayward, 1967)	(4) **0.40**	**0.60**				
*Vanessa carye* (Hübner, 1812)	**0.10**	**0.10**	**0.30**	**0.70**	(1) **0.42**	(5) **0.87**

Total	(65) 5.59	(10) 3.80	(10) 3.20	(1) 1.56	(21) 4.27	(38) 11.13

*Names are as per the Darwin Andean Butterfly Database (2010, http://www.andeanbutterflies.org/database.html).*

*Values in bold represent average number of individuals in a 15 min observation period from the 2008–2010 sampling. Values in parentheses represented number of records in collections consulted. No measures of variance are shown due to variation in sampling effort.*

**Table 1** shows the communities present in each habitat type. Altitudinal zonation is clear, with most species present in a relatively narrow altitudinal range (except the satyr *Vanessa carye*). Wetlands tend to show similar communities, but higher diversity and abundance than corresponding-altitude slope sites. Neoriparian habitats exhibited similar fauna, with higher abundance, as low-level wetlands. One notable exception is *Nabokovia faga* which was observed in neoriparian areas only (in corn fields) and never in natural habitats.

### HABITAT CLASSIFICATION

**FIGURE 2:**
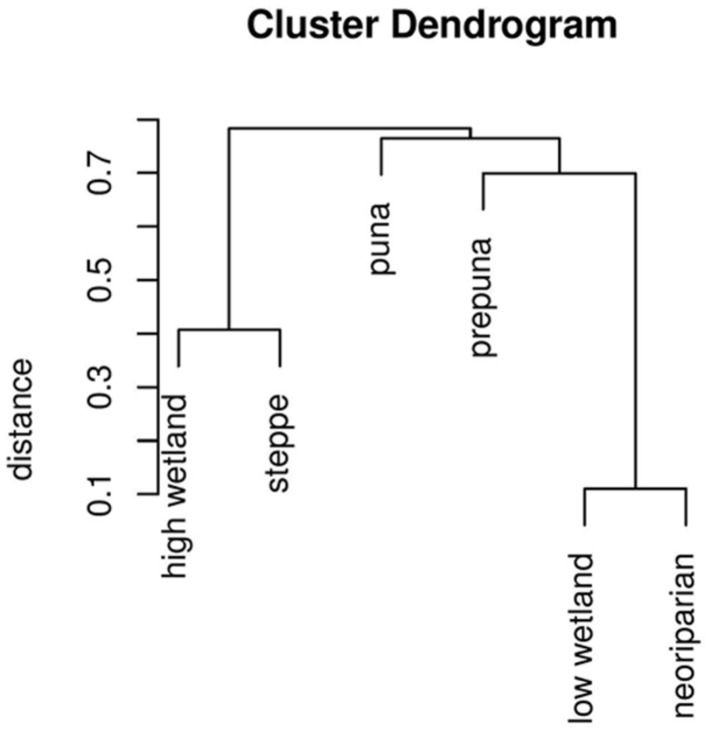
**Dendrogram of butterfly community similarity among habitats, illustrating the results of single-linkage cluster analyses based on the Morisita-Horn abundance-based similarity estimator.** Values on the *x*-axis associated with each node represent the difference between the sites connected by that node.

The dendrogram clustering habitats by similarity is shown in **Figure 2**. The closest similarity was between neoriparian and low-level wetland habitats, then between high-level wetland and Andean steppe habitats. Altitude explains much of the difference between butterfly communities, and differences between wetland and slope sites are greater at the more arid low-altitudes than at the high-altitudes where precipitation is more abundant.

### COMPARISON BETWEEN YEARS

**FIGURE 3:**
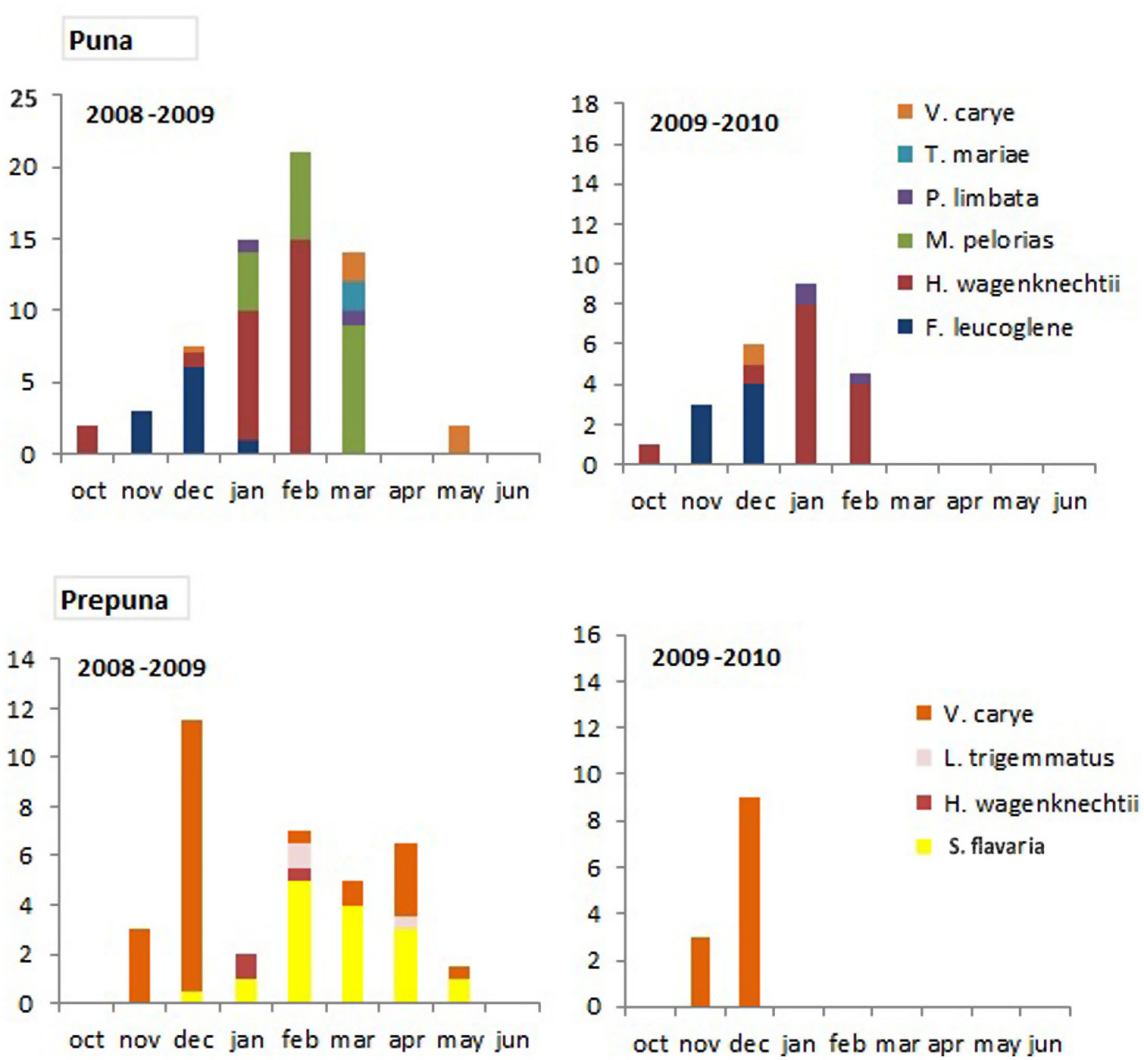
**Mean monthly abundance indices for all species in the puna and prepuna habitats in 2008–2009 and 2009–2010**.

Paired *t*-tests showed no significant differences in butterfly observations between the two seasons in high wetlands (*t*_351_ = 1.36, *p* = 0.16), low wetlands (*t*_87_ = 0.55; *p* = 0.58) or neoriparian habitats (*t*_107_ = 1.41, *p* = 0.17). Neither were there significant differences between years in butterfly sightings in the Andean steppe (*t*_98_ = 1.59, *p* = 0.11). However, differences approached significance in the puna (*t*_98_ = 1.63, *p* = 0.07) and were highly significant in the prepuna habitat (*t*_98_ = 2.62, *p* = 0.01). Butterfly abundance was much lower in 2009–2010 than in 2008–2009. Spring faunas were similar, but species active later in the summer after the monsoon precipitation were much less abundant. In particular, *V. carye* appeared to display two generations in 2008–2009 and only one in 2009–2010. Some species (e.g., *Strymon flavaria*, *Madeleinea pelorias*, *Tatochila mercedis macrodice*) were not seen at all in the second year (**Figure 3**).

## DISCUSSION

The results show strong altitudinal zonation matching the vegetation belts, as in a previous study (Despland et al., 2012) and show similarity between slope and wetland habitats at similar altitude. Our findings also demonstrate vulnerability to weather fluctuations in the more arid, low-altitude slope habitats. Indeed, during the dry year 2009–2010, no significant difference from the preceding year was observed in high-altitude or wetland habitats, but abundance was much lower in the low-altitude slope habitats, with several species failing to be recorded at all.

At high-altitude, *Palmaris penai, Faunula leucoglene*, and *Pierphulia rosea* were common in the steppe habitat and were also observed in wetlands. The first two show a narrow high abundance peak in spring, whereas *P. rosea* is seen in low numbers over a longer period during the summer (Despland et al., 2012). *Hylephila boulleti* and *Phulia nymphula* are high-altitude wetland specialists observed at all sites sampled. The sulfur *Colias flaveola blameyi* was only observed in one of the sampling sites (22°35′51.60′′S 68° 4′15.94′′W, see **Figure 1**). The genus *Colias* is still undergoing reorganization and hence the exact taxonomic status of these specimens in uncertain (Shapiro, 1989; Grieshuber and Lamas, 2007) — individuals have been placed in the Museo Nacional de Historia Natural for reference. *I. illimani* and *Pyrgus barrosi* are two high-altitude species that appear to have been rare historically (Peña, 1961; and see low numbers in collections in **Table 1**), but which have not been observed in recent years (Benyamini, personal communication). Our results confirm that the skipper *P. barrosi* is still present in the region, albeit at low abundance (see **Table 1**); however, the white *I. illimani* was not observed in any of the wetlands sampled, despite sampling in the same places and at the same times of year where it was observed in the past (Peña, 1959, 1964).

The Puna zone, among the slope habitats, contains the highest plant diversity and productivity, as well as the highest diversity of butterflies, including some overlap of higher- or lower-altitude species as well as some specialists: the blue *M. pelorias* was observed throughout the summer, generally associated with its larval host plant, the shrub *Adesmia* (Fabaceae; Benyamini, 1995), and *H. wagenknechtii* was observed hill-topping (Courtney and Shapiro, 1986a) during a few weeks in January.

At low-altitude, abundance and diversity were low in the prepuna habitats: these are the most arid of all habitats sampled. A peak of abundance of the nymphalid *V. carye* was observed early in the spring, and the hairstreak *S. flavaria* was observed throughout the summer. Wetland habitats showed a much higher diversity, including specialists like *Hylephila isonira* and *Pyrgus fides.*

The neoriparian habitats showed similar faunas to the low-altitude wetlands dominated by natural vegetation but with much higher abundance: these were dominated by the skipper *H. isonira* in spring, and numbers dropped gradually over the summer. As the summer progressed, the blue *Leptotes trigemmatus* become increasingly dominant, peaking in December–January. The hairstreak *N. faga* was observed in low numbers starting in March and increasing gradually during the autumn. Most low-altitude species are also found in neoriparian habitas (see **Table 1**); the notable exception being *S. flavaria,* which was seen only in prepuna habitats.

Butterflies are closely linked to plants, both as herbivores and pollinators, and previous work suggests that the altitudinal distribution of a butterfly might depend on that of its larval host plant (Brehm et al., 2003; Pyrcz et al., 2009) and hence that altitudinal patterns in butterfly diversity might match those of plants. The larval forms and host plants have been described for some of the species (Courtney and Shapiro, 1986b; Shapiro and Courtney, 1986; Benyamini, 1995; Beccaloni et al., 2008), and these fit with the observations recorded here. For instance, *H. isonira* larvae develop on a weedy grass (*Distichlis* spp.), which was common in both low-altitude wetlands and neoriparian habitats, but was not observed at any of the other sites.

These associations can also help explain why some low-altitude butterfly species have adjusted to the agricultural matrix of the oasis, while others have not. In the neoriparian habitat, all the butterflies were frequently observed nectar-feeding on alfalfa flowers, suggesting that this year-round food source constitutes an important resource for these insects. In a warm arid climate like that of the salar de Atacama, irrigation prolongs the growing season and hence provides good habitat for those butterflies that can reproduce on plants available within the agricultural matrix. For instance, *V. carye* larvae were observed on introduced *Malva* spp. ornamental plants. Both the lycaenids *N. faga* and *L. trigemmatus* are legume feeders that can use alfalfa as a larval host (Benyamini, 1995). The present study suggests that alfalfa is a key resource for butterflies in agricultural areas, both as a nectar source for adults and a larval host plant. However, studies with a North American legume feeding lycaenid that has also shifted to alfalfa show that this plant is attractive to butterflies mainly because it provides nectar source as well as an oviposition substrate, but that larvae perform less well than they do on native food plants, reaching smaller adult sizes (Forister et al., 2009). Indeed, *L. trigemmatus* specimens collected in the neoriparian zone in the present study appeared smaller than museum records – whether this is linked to larval host plant is unknown but would merit further attention, due to the implications for assessing habitat quality of the agricultural matrix.

Studies in arid zones of Argentina show a similar shift to exotics in wetlands, where modification of the vegetation is greatest: in neoriparian habitats, butterflies are generally much more abundant than in the adjacent native vegetation steppe, the species mix is very different and most of the species reproduce on exotic host plants (Shapiro, 2002). However, the Argentine work only compared similar-elevation slope habitats with heavily impacted wetland areas, and did not include native wetland vegetation because it is almost inexistant in that region. We were able to include wetlands still dominated by native plants and show that their butterfly faunas more closely resemble those of neoriparian areas than of prepuna habitats (see **Figure 2**). The ability to use either exotic or ruderal plants present in neoriparian sites seems key. In the heavily impacted mediterranean landscapes of California, 34% of native butterflies use introduced plants as larval hosts (Graves and Shapiro, 2003): geographically widespread generalists are more likely to colonize novel exotic hosts and species that are not able to shift to exotic hosts are more likely to go extinct (Jahner et al., 2011).

The region around the salar de Atacama has a millennia-long history of agriculture, including introduction of crop plants and irrigation in lower altitude oases and riparian areas, and grazing livestock on the Andean slope (Villagrán and Castro, 2003; Nuñez, 2007). As a result, the landscape has been shaped by human activities, especially in the oases which are dominated by a mosaic of alfalfa pastures, corn and other crops and abandoned lots colonized by ruderal plants (Gutierrez et al., 1998). The association of alfalfa and ruderal native plants in our study site is characteristic of this oasis biome (Gutierrez et al., 1998). Traditional agriculture in the Atacama provides habitat for certain butterfly species that might otherwise be less common. At a time where this traditional agriculture is gradually being abandoned in favor of employment in mining or tourism (RIDES, 2003), the habitat value of the traditional agricultural landscape needs to be considered (New et al., 1995).

However, the ability to acclimate to human-modified landscapes is clearly variable among species. The lycaenids *N. faga* and *M. pelorias* are both documented as *Adesmia* (Fabaceae) feeders found in the puna vegetation belt (Benyamini, 1995). In our study, *N. faga* was not observed in the native puna sites, but has been able to shift to the agricultural matrix by using alfalfa as an alternate larval host. Conversely, *M. pelorias* was still only seen in the native puna habitat. Similarly, *S. flavaria* was only observed in prepuna sites. Neither of these species was observed at all in the lower precipitation sampling year, suggesting that they are vulnerable to weather fluctuations. The diapause strategies of these butterflies are unknown: some desert species can remain in diapause during dry years, but this does not seem to occur in Andean lycaenids (Powell, 1986; Johnson and Coates, 1999). In general, little is known about the ecology and life history for most of these butterflies, which makes it difficult to draw inferences about their vulnerability to environmental change.

The puna plant community is vulnerable, and overgrazing by livestock can be a significant conservation concern (RIDES, 2003). In particular, fabaceous shrubs in the genus *Adesmia* appear to be a key resource for herbivores in puna ecosystems (Squeo et al., 2006a): they are hosts for a variety of Lycaenid (Benyamini, 1995) and Pierid (Shapiro, 1992; Squeo et al., 2006a) caterpillars and are heavily used by livestock brought up to the puna belt to graze (Villagrán and Castro, 2003; Squeo et al., 2006b). In central Chile, several butterfly species have been extirpated due to unregulated livestock grazing on *Adesmia* host plants (Johnson, 1999), and here is concern that the same thing might be occurring in the arid North. For instance, *Pseudolucia oligocyanea* (Lycaenidae) is known from a few specimens collected in the puna belt above the salar de Atacama in the 1950s (Benyamini, 1995). It was collected again in the region in 1999 (Johnson, 2000), but not since then (Benyamini, personal communication) and was not seen in the present study. The absence of *I. illimani* from the high-altitude wetland sites raises a similar concern. The already arid landscape is becoming increasingly dry (Benyamini, 1995; RIDES, 2003), putting additional pressure on vulnerable and poorly understood ecosystems.

## Conflict of Interest Statement

The author declares that the research was conducted in the absence of any commercial or financial relationships that could be construed as a potential conflict of interest.
